# Therapeutics

**Published:** 1876-03

**Authors:** 


### IV. THERAPEUTICS.

1. *Physiological Antidotes to Opium.* PROF. J. W. HOLLAND. (*Louisville Medical News*, 1876.)

Two cases treated by the Professor prompted his article. To these victims of opium, everything regarded *au fait* had been administered. Emetics, stomach pump, atropia, coffee, *et id omne genus*, failed in accomplishing satisfactory results. Electricity applied in accordance with instructions of the books, yielded no hopeful returns. The battery was applied through the neck, brows, chest, from the neck to the epigastrium, from the neck to the feet, from hand to hand, and from the nape to the forehead. Some of these various applications produced more effects on the breathing than the others; and careful comparison convinced the Professor that the second place of application—viz., *through the brows*—possessed the greatest stimulating effect on respiration. Application of the electrodes upon these points of exit of the *supra-orbital nerves* was the finally adopted plan of treatment which was followed by a return to consciousness. Alternately shifting the electrodes, after keeping them in this position for a time, to some other portion of the body and then back again to the brows, was followed by better stimulating effects than the constant application produced. The writer thinks that it is far from being a *proven* fact that atropia is the antidote to opium.

(The editors desire to state that Pres. Freer of Rush Medical College has now under consideration and experimentation the theory that *artificial respiration* is, *par excellence*, the physiological antidote to opium narcosis. When he reaches a reliable conclusion he promises the JOURNAL AND EXAMINER an article on the subject.)

2. *Hypodermic Alimentation.* KRUEG. (*Wiener Med. Wochenschrift. Times and Gaz.*, Sept., 1875.)

Sustentation of life by placing food beneath the skin has excited incredulity in the minds of physiologists, "because," they say, "peptones can not be formed in that location." The subject of this observation was an Hungarian who had been in an asylum at Ober-Doebling since 1868; he had frequently attempted self-destruction by refusing all food, so that at one time he had to be fed regularly for 27 consecutive months. Sometimes he would relent and eat voluntarily. On January 18, 1875, he began again to absolutely refuse food, and continued so, excepting one day, till Jan. 24, when it was resolved to again resort to the tube. In using the latter, however, all attempts were foiled by incoercible vomiting, suffering, breathlessness and cyanosis. As ten days had elapsed without food, with the exception of one day, hypodermic injections of olive oil were determined upon. A syringe of 15 cubic centimetres (about three ounces) capacity was used, and one to two syringefuls were injected daily for the ensuing twenty days. The patient thus received from three to six ounces daily of olive oil. Each syringeful was put into from two to five different apertures. Sometimes the foot was selected as the seat of puncture, at other times the belly and sides. The canula of an ordinary hypodermic syringe was used, between which and the syringe was a thin caoutchouc tube to prevent the patient's deranging the working of the apparatus. Occasionally, *after* the 29 consecutive days of fasting—during the last 20 of which hypodermic injections of olive oil were used—the patient would take food and then he would again refuse to eat, when the injections were resumed. At length his opposition to food was abandoned and he ate in a natural way, the experiment lasting about two months altogether.

3. *Another Case of Hypodermic Alimentation.* PROF. WHITTAKER. (*The Clinic*, Jan. 22, 1876.)

The patient, a young woman of 20 years, was first seen Nov. 3, 1875. Five years before admission she suddenly experienced a sharp epigastric pain, whilst actively exercising. This pain had persisted ever since, being aggravated by food. Five weeks prior to entering hospital she had suffered from vomiting. Nothing could be retained excepting the blandest food, and even that was sometimes promptly vomited. This condition obtained till Jan. 6, 1876, when the case was thus recorded: marked emaciation, exceedingly feeble pulse, almost imperceptible, high temperature (not stated how high), pains in the back and head, in the afternoon and night the delirium of inanition, complete exhaustion, death imminent. Hypodermic injections of a teaspoonful of milk, alternated with beef extract, were given every two hours. These injections were continued from the 6th to the 9th inst., inclusive. Under their use the patient's "temperature declined, the pulse became fuller and stronger, and the delirium and pain disappeared." The injections were continued till the 12th inst., when the patient was able to eat without pain or nausea. During these few days,

68 injections in different parts of the body were administered. During the last two days, cod liver oil was substituted for milk and beef extract. Only two abscesses were developed among all the 68 punctures, and those two were caused by the milk. The oil injections were absolutely painless, the oil being injected slowly. On one day as much as four ounces of oil were used. According to Prof. Whittaker the absorption of the cod oil is facilitated by its contained bile which is detectable by sulphuric acid. In the alimentary canal, oils are absorbed largely by the blood-vessels and not exclusively by the lacteals. The presence of bile is necessary to this absorption—a fact proven by physiologists. Hence cod liver oil subcutaneously injected is absorbed by the capillaries in its neighborhood precisely in the same manner as by the sub-mucous blood-vessels of the small intestine.

4. *A New Use for Carbolic Acid—Opening Abscesses without Pain.* Dr. BERGONZINI, of Bologna. (*Revista di Bologna*, 1875.)

Put on the skin for three or five minutes, a solution of two parts of carbolic acid to one part of glycerine. If the skin be previously inflamed (as is ordinarily the case in abscesses following great inflammation) a too protracted contact of the anæsthesiant liquid must be avoided. Dr. Bergonzini, who has used this very simple means with success, thinks that it can be utilized in autoplastic operations as well as in superficial neuralgias.

5. *Coffee as a Preventive of Malarial Diseases.* PROF. J. W. HOLLAND, M.D. (*Louisville Med. News.*)

The writer calls attention to the instinctive desire for coffee experienced by well persons and those sick from malarial causes, residing in malarial districts of the country. The design of his writing the article is to elicit facts relative to this subject. His own experience led him to suppose that coffee drinking protected him in a great measure from the effects of malaria. The Confederate army in our late war suffered from the lack of coffee, while the Federal army was measurably free from the suffering noticed in the former, because it was wisely supplied with an abundance of material for this beverage. The fact is elicited from a communication to the writer from a student from the Indian Territory, on Arkansas river, six miles west of Muskogee, an excessively malarial district, that the Indians call for coffee as the first article of purchase at the trading-post, when they take in their furs for exchange. They can not get along without it. Patients in this section of country, sick from malarial causes, recover quicker when allowed the much-desired coffee during their illness. Another student, from Pekin, Indiana, says that coffee is regarded as a prophylactic against palustral diseases. Coffee drinking in his country is very common. The more decidedly malarial the district, the more and stronger the coffee used.

(Malarial poison, whatever it is, undoubtedly is a "depressant to the nervous system," and coffee acts as Prof. Gubler teaches that quinine acts, *not* by *destroying* the disease germ, but by *stimulating* the system, producing a predominance of the latter effect. Caffeine and its consort, theine, are wonderful stimulators, and coffee produces much the same effect as quinia does in malarial parts of the country. It is easy to understand the instinctive longing for coffee in persons residing in districts rich in malaria.—Ed.)

6. *Senna in Remittent Fever.* (*The Canada Lancet*, Feb., 1876.)

The writer strongly recommends this drug to relieve the symptoms pointing to cerebral disturbance. The chief indications for its use are cephalalgia, pain, confusion of thought and delirium—one or all. The open or confined condition of the bowels is of no moment; if the head symptoms supervene, senna purgation will relieve them, lessen the febrile movement as indicated by the thermometer, and increase the duration of the remission. Quinine can then be more advantageously administered.

7. *Successful Restoration from Chloroform Narcosis by Nélaton's Method.* Eugene Smith, M.D. (*Detroit Review of Medicine.*)

The patient was a girl seven years old, and after the operation (for strabismus) was finished, she suddenly ceased breathing, and "there was no radial or carotid pulse." The patient was held up by the ankles, the head hanging down, and artificial respiration made, while she was held in that position. After three or four minutes "there was a feeble gasp—and after awhile another, and then another," and afterwards the breathing was restored. The breathing ceased a second time, shortly after laying her down, and a second restoration, similar to the first, was similarly effected. For the third time she passed through the same terrible experience, in a few moments subsequently, and the same means restored her again, just as they were about to give her up, because this period was the longest of the three, and the doctor supposed the patient dead. The third restoration was followed immediately by vomiting, and complete consciousness quickly followed.

8. *Poisoning by Goat's Milk.* (*Gazette Obstét. et Gynécol.*, Jan. 20, 1876.)

The German and Italian journals report the history of a small epidemic in the environs of Rome, whose source is somewhat curious. A great number of the inhabitants were seized with gastro-intestinal irritation, characterized by diarrhœa, vomitings, and intense thirst, a notable diminution in the temperature, and a frequency of the pulse.

After investigation, the physicians suspected the goat's milk which is in general use in the country. The animals were examined by the veterinary surgeon and pronounced healthy. The milk was analyzed, as well as the dejections from the patients, and not a single trace of metallic poison was found.

Suspicion then rested upon the ordinary pasturage of the goats, and there were found four plants more or less poisonous, viz.: *Clematis Vitelba*, *Conium Maculatum*, *Colchicum Autumnale*, and *Plumbago Europœa.* The matters vomited and the milk (analyzed anew) presented the chemical reactions characteristic of *colchicine.*

9. *A New Means of Distinguishing Apparent from Real Death.* (*El. Pabellon Médico.*)

It consists in injecting a drop of ammonia beneath the skin; if death is certain, this drop will produce no effect whatever, or almost none; if life still be present, a red spot will appear at the point of injection.

10. *Articular Rheumatism Treated by Salicylic Acid.* DR. STRICKER. (*Allg. Med. Zeitung*, 1876, No. 8.)

The author reports fourteen cases of articular rheumatism which, in Traube's clinic, were treated with salicylic acid. "Within forty-eight hours all the patients not merely recovered of the increase of temperature, but also were entirely relieved of the local symptoms, *i. e.*, the swelling, redness, and particularly the pain and tenderness of the joints." He, of course, considers the salicylic acid to be the most efficient, perhaps a specific, remedy for the acute rheumatism of joints. But he insists upon administering a pure acid, which forms perfectly white crystalline needles and with water and alcohol makes a perfectly clear solution. The pure acid can be given in pretty large doses without any injury to the digestive organs; the caustic property attributed to the salicylic acid the author claims to be due to impurities. He gave it in powders, one-half to one gramme each, every hour, until the diseased joints could be moved without pain; for this purpose no more than fifteen nor any less than five grammes were required; the larger doses produced a ringing in the ears and dullness of hearing (like quinine.)

11. *On the Action of Scilla.* HUSEMANN. (*Allg. Med. Zeitung*, 1876, No. 8.)

The author expresses his opinion based on numerous experiments on the action of the scilla as follows: The extractum scillæ, prepared after the receipt of the German pharmacopœia, is a very reliable preparation, and acts upon the innervation of the heart exactly in the same way as digitalis; its diuretic action must be attributed to the increased arterial pressure as the natural consequence of its action on the heart, for it has no stimulating nor irritating influence on the kidneys; it is not an antipyretic remedy; on the contrary, in smaller and larger doses, it always causes an increase of the temperature of the body.

